# Analysis of mixtures using next generation sequencing of mitochondrial DNA hypervariable regions

**DOI:** 10.3325/cmj.2015.56.208

**Published:** 2015-06

**Authors:** Hanna Kim, Henry A. Erlich, Cassandra D. Calloway

**Affiliations:** 1 Center for Genetics, Children’s Hospital Oakland Research Institute, Oakland, CA, USA; 2 Graduate Studies in Forensic Science, University of California, Davis, CA, USA

## Abstract

**Aim:**

To apply massively parallel and clonal sequencing (next generation sequencing or NGS) to the analysis of forensic mixed samples.

**Methods:**

A duplex polymerase chain reaction (PCR) assay targeting the mitochondrial DNA (mtDNA) hypervariable regions I/II (HVI/HVII) was developed for NGS analysis on the Roche 454 GS Junior instrument. Eight sets of multiplex identifier-tagged 454 fusion primers were used in a combinatorial approach for amplification and deep sequencing of up to 64 samples in parallel.

**Results:**

This assay was shown to be highly sensitive for sequencing limited DNA amounts ( ~ 100 mtDNA copies) and analyzing contrived and biological mixtures with low level variants ( ~ 1%) as well as “complex” mixtures (≥3 contributors). PCR artifact “hybrid” sequences generated by jumping PCR or template switching were observed at a low level (<2%) in the analysis of mixed samples but could be eliminated by reducing the PCR cycle number.

**Conclusion:**

This study demonstrates the power of NGS technologies targeting the mtDNA HVI/HVII regions for analysis of challenging forensic samples, such as mixtures and specimens with limited DNA.

Limited and mixed DNA samples are often encountered in forensic cases and pose both technical and interpretation challenges. The highly polymorphic hypervariable regions I/II (HVI/II) of the mitochondrial genome are often successfully used to analyze limited and/or degraded DNA samples (1). However, there are some limitations to the current standard approaches used for mitochondrial DNA (mtDNA) sequence analysis when mixtures are encountered. In a five year retrospective study of mtDNA analysis of 691 casework hair samples, a mixture of mtDNA sequences attributed to a secondary source was observed in 8.7% of the hairs and sequence heteroplasmy was observed in 11.7% of the cases (2). While approaches that use capillary electrophoresis technologies for Sanger sequencing of mtDNA polymorphic regions allow for detection of mixtures, they do not allow for resolving individual sequences in a mixture (3-10). Mitochondrial DNA markers are ideal targets for detecting mixtures since, with few exceptions, a single sequence per contributor is the expected result due to its haploid nature. However, unlike short tandem repeats (STRs), peak areas or heights in sequence electropherograms are not necessarily indicative of the amount of DNA contributed to a mixture (9,11,12). As a result, peak height ratios for two bases cannot be used to determine the relative proportions of components of a mixture for mtDNA Sanger sequencing analysis. For this reason, Sanger sequencing does not allow for determining the individual mtDNA sequence haplotypes of mixed samples. Therefore, when mixed base calls are encountered during mtDNA Sanger sequence analysis of forensic specimens, most forensic laboratories choose not to interpret the result and categorize mtDNA mixture results as inconclusive for reporting purposes (13). Furthermore, Sanger sequencing cannot detect minor components present at less than 10% in a DNA mixture (9,12).

The 454 genome sequencing technology is a scalable, clonal, and highly parallel pyrosequencing system that can be used for de novo sequencing of small whole genomes or direct sequencing of DNA products generated by polymerase chain reaction (PCR). The technology uses emulsion PCR (emPCR) to amplify a single DNA sequence to 10 million identical copies. The “clonal sequencing” aspect of the technology enables separation of individual components of a mixture as well as analysis of highly degraded DNA. The clonal sequencing approach used with the 454 GS technology and other next-generation sequencing (NGS) technologies provides a digital readout of the number of reads or individual sequences allowing for a quantitative determination of the components in a mixture (14). Recently, the potential value of using NGS technologies for forensic applications has been demonstrated (15-18). This article aims to describe a highly sensitive NGS method that uses PCR for targeted enrichment of the HVI/HVII regions of mtDNA for resolving simple and complex mixtures as well as detecting low levels of heteroplasmy.

## Materials and methods

The study was conducted between September 2012 and January 2014 at Children’s Hospital Oakland Research Institute, Genetics Department, and was approved by the Children’s Hospital & Research Center Oakland Institutional Review Board.

### 454 library preparation: HVI/HVII PCR targeted enrichment

**Figure 1:**
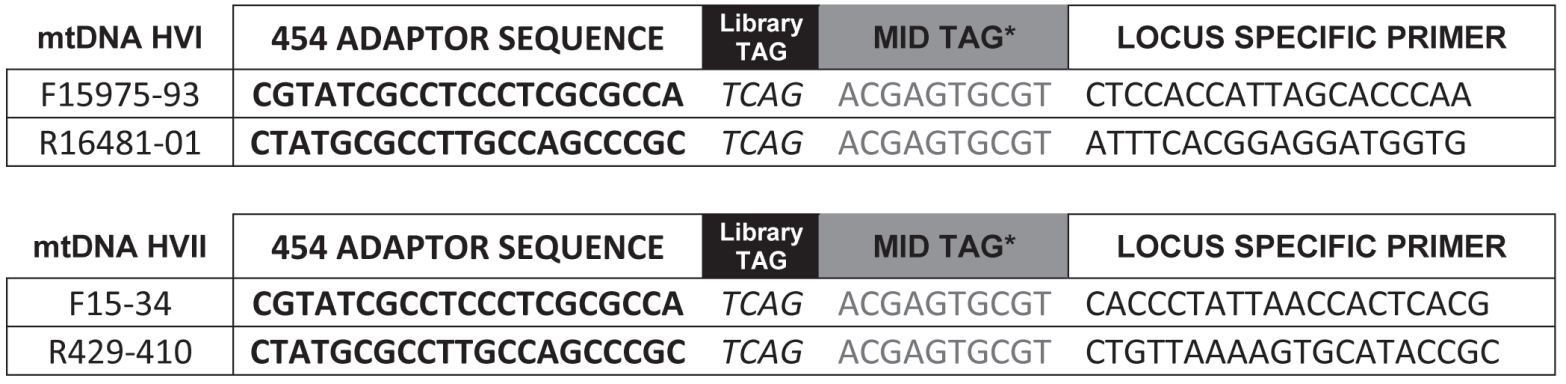
Hypervariable region I/II (HVI/II) I forward and reverse fusion primers. A total of four primers comprised of a duplex primer set targeting HVI and HVII. Each primer consists of the 454 Adaptor Sequence (bold), Library Key Sequence (italics), multiplex identifier (MID)-tag sequence (gray), and the forward or reverse HVI/HVII locus specific primer sequence (19). The numbering used for the primer names corresponds to the revised Cambridge reference sequence (rCRS) (20). The MID sequences (gray) are tagged with published MID sequences 1 through 8 to create eight sets of primers per targeted region (Supplementary Figure 1 (Supplementary Figure 1)).

The highly polymorphic HVI and HVII regions of the mitochondrial genome were enriched from genomic DNA samples using a duplex PCR. Mitochondrial DNA HVI/HVII duplex primer sequences described previously in Gabriel et al (19) were modified to incorporate the 454 sequencing primer, library key, and unique multiplex identifier (MID) sequence necessary for subsequent emPCR, pyrosequencing, and sample identification during the data analysis. Each sample was amplified using a unique MID-tagged 454 HVI/HVII fusion primer set. A set of HVI/HVII fusion primer sequences with MID tag 1 are shown in the Figure 1. Unique 10 base MID tags are used as sample identifiers in order to pool and sequence multiple samples in a single 454 sequencing run with each sample being “tagged” or “barcoded” with a different MID tag. The eight sets of MID tags used for the fusion primer sets are provided in Supplementary Table 1 (web Supplementary Table 1). The eight sets of MID-tagged fusion primers were designed to be used in a combinatorial approach to generate 64 different combinations of forward and reverse MID-tagged HVI/HVII PCR products (Supplementary Figure 1 (Supplementary Figure 1)). This combinatorial approach was utilized in order to reduce the number of PCR primers required.

Each sample was amplified in a 50 µL PCR reaction containing 1.2X GeneAmp^®^ PCR Buffer II without MgCl2 (Life Technologies, Foster City, CA, USA), 2.4 mM MgCl_2_, 0.2 mM each dNTP (Roche Life Sciences, Indianapolis, IN, USA), 0.25 U/µL EagleTaq DNA Polymerase (Roche Custom Biotech, Indianapolis, IN, USA), and 0.3 µM each primer. Both HVI (15975-16501) and HVII (15-410) regions were amplified in a single tube using a set of MID-tagged fusion primers. All reactions were performed in GeneAmp^®^ PCR Systems 9600 thermal cycler (Applied Biosystems, Carlsbad, CA, USA) using the following cycling conditions: a 14-minute 94°C activation cycle; 34 cycles of amplification at 94°C for 15 seconds, 65°C for 30 seconds, and 72°C for 30 seconds; and a final 10-minute 72°C extension cycle. PCR products were analyzed on 1.5% agarose gels in 1X Tris-Acetate-EDTA electrophoresis buffer (TAE) stained with 0.5 µg/mL ethidium bromide to confirm successful amplification of the target regions and to assess primer dimer and PCR specificity.

### 454 library preparation: PCR product quantification and small fragment removal

Each PCR product was quantified using Quant-iT PicoGreen^®^ dsDNA Assay kit (Life Technologies) to determine the DNA amount in copy number for sample normalization and subsequent library pooling. An approximately equal copy number of each amplified sample was combined into a single library pool. Small fragment DNA or primer dimers were removed from the pooled library using Agencourt AMPure XP (Beckman Coulter Inc., Pasadena, CA, USA) with 1:1 AMPure to DNA sample volumetric ratio. The Agilent DNA 1000 kit (Agilent Technologies, Santa Clara, CA, USA) was used following manufacturer’s protocol in order to compare the library pool before and after AMPure purification to confirm the removal of primer dimers. The concentration of the purified library pool was estimated using Quant-iT PicoGreen^®^ dsDNA kit (Life Technologies) or Library Quantification Kit - 454 FLX (Kapa Biosystems, Wilmington, MA, USA) to estimate the DNA copy number of the library pool.

### 454 emulsion PCR and NGS using the GS Jr

The quantified and purified library pool was then diluted with modified TE-4 (10 mM Tris, 0.1 mM EDTA, pH 8.0) to the concentration of 4 × 10^5^ molecules/uL in order to add a total of 10 uL to achieve a 0.4:1 DNA to bead ratio in emPCR. The optimal DNA to bead ratio was determined to be 0.4:1 based on the number of high quality pass filter reads. Targeting the optimal DNA:bead ratio is critical to avoid the incorporation of more than one molecule of DNA per bead or insufficient bead recovery. The finalized library pool was amplified using the GS Junior Titanium emPCR Kit (Lib-A) (Roche Life Sciences) following the manufacturer’s protocol. All successfully prepared libraries were sequenced on the 454 GS Junior System (454 Life Sciences, Branfod, CT, USA). Amplicon Variant Analyzer (AVA) was used to analyze the 454 sequence data and identify SNP variants compared to the revised Cambridge sequence (rCRS) (20).

### Sample preparation and experimental designs: sensitivity

*Sensitivity and concordance studies.* A sensitivity study was conducted to determine the limits of the assay as follows. The mtDNA copy number and nDNA concentration of a control DNA sample K562 High Molecular Weight, (Promega, Madison, WI, USA) was quantified using a mt:nu qPCR assay and serially diluted (21). Thirteen dilutions varying in starting mtDNA copy number (1 000 000 to 1 mtDNA copy) and nDNA input amount (1 ng to 0.001 pg) were amplified (Table 1). The 13 libraries were then normalized for amplicon copy number estimated using the Qubit^®^ dsDNA HS Assay Kit (Life Technologies), combined into a single library pool, and sequenced using the 454 GS Jr. To determine the accuracy of the assay, 454 HVI/HVII sequence data were generated and analyzed from previously collected blood derived DNA samples from four population groups (45 African American, 43 Caucasian, 40 Hispanic, and 45 Japanese) (22). Sanger sequencing results were available for comparison to the 454 NGS data (23).

**Table 1:** Number of sequence reads obtained from varying DNA input amounts

DNA amount	mtDNA copy number	Number of 454 reads					
		Hypervariable region I			Hypervariable region II		
		forward	reverse	total	forward	reverse	total
1 ng	~ 1 000 000	56	116	172	189	110	299
500 pg	500000	43	103	146	174	94	268
100 pg	100000	61	122	183	161	97	258
50 pg	50000	63	145	208	177	105	282
10 pg	10000	56	88	144	130	105	235
5 pg	5000	91	156	247	138	121	259
1 pg	1000	56	130	186	104	78	182
0.5 pg	500	80	131	211	195	115	310
0.1 pg	100	19	37	56	66	45	111
0.05 pg	50	24	48	72	59	52	111
0.01 pg	10	0	5	5	5	0	5
0.005 pg	5	0	4	4	4	0	4
0.001 pg	1	3	0	3	6	4	10

*Contrived and biological mixture studies.* Mixture studies (two and three person mixtures) were conducted to determine the sensitivity of the assay for detecting and resolving the minor sequences in a mixed specimen. Two Coriell Cell line DNA samples (Coriell Institute, Camden, NJ, USA), differing at 10 base positions (7 in HVII and 3 in HVI) determined previously using Sanger sequencing were mixed together at various ratios based on the mtDNA copy number. The mtDNA copy number was determined using a mt:nu qPCR assay described by Timken et al (21). The two DNA samples were mixed at the following ratios and were then amplified using the 454 HVI/HVII NGS PCR assay and sequenced using the 454 GS Jr: 100:0, 99.9:0.1, 99.75:0.25, 99.5:0.5, 99:1, 97.5:2.5, 95:5, 90:10, 75:25, 50:50, 25:75, 10:90, 5:95, 2.5, 97.5, 1:99, 0.5:99.5, 0.25:99.75, 0.1:99.9, and 0:100. A complex mixture sample was prepared by combining DNA based on the mtDNA copy number of three individuals and the HVI/HVII amplicon library was prepared following the above described method and sequenced using the 454 GS Jr. instrument. To study the effect of cycle number on generation of jumping PCR hybrids with the 454 HVI/HVII NGS assay, a 50:50 mixture of two Coriell Cell line DNA samples (Coriell Institute), was amplified at varying cycle numbers ranging from 34 to 24 cycles in 2 cycle increments. DNA from a buccal swab, blood, and five hairs from a monozygotic twin pair previously identified as heteroplasmic in at least one tissue was analyzed using the HVI/HVII NGS assay. DNA libraries were prepared by amplifying ~ 100 pg DNA from each sample and sequenced using the 454 HVI/HVII NGS assay (24,25). The HV+ 5plex and 10plex typing results for these samples were available for comparison (unpublished data).

## Results

### Sensitivity and reproducibility of the assay

**Figure 2:**
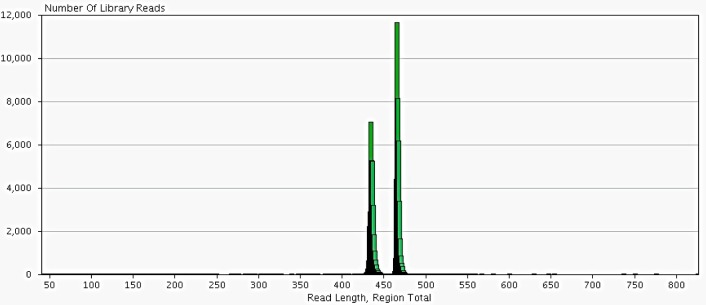
Distribution of reads for 454 HVI/HVII NGS Assay. The read length distribution of a 454 hypervariable region I/II (HVI/HVII) sequencing run reported by GS Run Browser software (454 Life Sciences, Bradford, CT, USA). The shorter peak of 450 bp corresponds to the HVII library amplicons (415 bp HVII region) and the 475 bp product to amplified HVI libraries (444 bp HVI region). The absence of any peak below 430 bp indicates the effective removal of primer dimers.

A sensitivity study was conducted to determine the minimum starting DNA amount of the 454 HVI/HVII NGS assay as well as to explore the stochastic effects in the PCR amplification step of the assay. The HVI/HVII 454 sequencing assay was shown to be highly sensitive for limited input of genomic DNA. Amplification and sequencing was successful for samples with DNA amounts as low as ~ 1 pg genomic DNA or 100-500 mtDNA copies (Table 1). DNA amounts ranging from 0.5 pg-1 ng resulted in an average of 261 combined reads, ranging from 182-310 reads (Table 1). The number of reads decreased 2-fold with samples amplified with 0.1 pg and 0.05 pg of input DNA relative to higher input amounts. Also, DNA starting amounts lower than 0.05 pg resulted in an extremely low number of reads, often unidirectional (Table 1). Based on these results, the assay sensitivity was determined to be 0.5 pg or ~ 500 copies. However, the limit of starting DNA amount of the assay could potentially be lowered to 0.1 pg or 0.05 pg by elimination of the dilution process after the amplification of the low copy number samples along with increasing the amount of PCR product added for the sequencing for those specific samples. This sensitivity study demonstrated that NGS results can be obtained from lower amounts of DNA than previously reported or recommended by the manufacturer (pg amounts compared to ng or µg amounts). Also, the assay was very specific and did not produce any fragment reads outside of the targeted HVI/HVII regions or short fragments (primer dimer) (Figure 2). Only reads corresponding to the sizes of the HVI and HVII amplicons were observed.

### Concordance study on population samples

The sequence data of population samples produced from the 454 HVI/HVII NGS assay were concordant with Sanger sequencing results for the HVI and HVII regions outside of homopolymer C stretch regions (positions 16180-16195 and 286-315, respectively). The sequences generated by the 454 HVI/HVII NGS assay showed concordance with the Sanger sequencing results at all base substitution positions (point mutations) when a 10% threshold was applied to the data analyzed using the 454 AVA software. A 10% threshold was applied to account for the lower sensitivity of Sanger sequencing for minor base detection (10%-15%) compared to 454 sequencing (1%-3%). Minor base differences were observed at some base positions at levels below the 10% threshold with 454 sequencing. These minor base differences observed at very low frequency (<1%) can be attributed to PCR or sequencing error or heteroplasmy; further analysis is needed in order to distinguish true signal from noise.

As expected, 454 pyro-sequencing errors were observed as insertion/deletions (IN/DEL) above the 10% threshold within the sequence surrounding the two homopolymer C stretches in the HVI/HVII regions, which resulted in sequence alignment errors using the AVA software. Typically, these IN/DEL sequencing errors were observed in only a single direction and/or the base calls for a given position were imbalanced. Specifically, pyro-sequencing errors and/or length heteroplasmy in the HVI region spanning base positions 16180-16195 (5′-AAAACCCCCTCCCCAT-3′) resulted in alignment issues using the AVA software. An A/C mutation at position 16183 and any C/T transition observed between base positions 16184 to 16193 of this region was reported as a combination of IN/DEL of A/C or C/T, with imbalanced frequencies between read directions. The SNPs in the homopolymer region were detected, but were not reported correctly due to alignment issues from homopolymer pyro-sequencing errors or potentially length heteroplasmy using the 454 AVA software.

Additionally, pyro-sequencing errors and/or length heteroplasmy within the homopolymer region surrounding the C-stretch in the HVII region spanning positions 286- 315 (5′-AAAAAATTTCCACCAAACCCCCCCTCCCCC-3′) were observed. Within this region, an A deletion was sometimes reported at base position 291, and position 295 was reported as a mixture of C/T followed by C deletion both with directionally imbalanced frequencies using 454 AVA software. These observations can be partially explained as a sequence alignment issue for the specific regions with homopolymers within the HVI/HVII sequences using the 454 sequencing platform as it is well established that pyro-sequencing has a relatively high error rate in homopolymer regions (26).

### Quantitative analysis of mixture sequences and PCR artifacts

We conducted a two person mixture study to determine the sensitivity of the 454 HVI/HVII NGS assay for detecting minor components in a mixture. 454 HVI/HVII sequencing results are presented in Table 2 for a subset of the mixture ratios. The minor and major sequences were detected using 454 NGS sequencing at all mixed based positions for samples mixed at a 2.5% ratio and above. For the 1% mixture ratio with COR110 as the minor sequence, the minor base was detected for all mixed based positions except position 150 in the HVII region with a 370 read depth. For the 1% mixture ratio with COR073 as the minor sequence, the minor bases at all mixed base positions were not detected in HVII but were in HVI. However, only 274 reads were obtained for the HVII region (average 1.7 reads per base for the minor sequence) compared to 516 reads for HVI region (average 3.0 reads per base for the minor sequence) for this sample. The minor contributor sequence in a 1% mixture would not be expected to be reliably detected with such low read coverage of 1-3 reads for the minor contributor sequence with a low depth of coverage (250-500). To reliably detect the minor sequence in a 1% mixture, greater than 1000 read coverage would be needed. An approximately 20%-40% difference from the expected mixture ratio was observed in each experimental mixture sample, with the number of reads for one sample in the mixture (COR110) consistently being under-represented. Based on these results, the mt:nu qPCR most likely overestimated the mtDNA copy number of C110 and thus the actual mixture ratio could have been skewed from the expected mixture ratio. These results show that NGS is a highly sensitive method for detecting minor components in a mixture (2.5% compared to 10%-15% for Sanger sequencing) and quantitative as a digital read-out can be reported by counting the number of sequence reads corresponding to the individual components.

**Table 2:** Minor base frequency in two person mixture

	Hypervariable region II								Hypervariable region I			
Expected frequency (%)	observed base frequency (%)							no. of reads	observed base frequency (%)			no. of reads
	94	150	152	189	194	200	228		16093	16327	16362	
**25**	17.1	17.1	18.1	18.1	17.31	17.31	17.31	387	20.5	21.51	22.35	595
**10**	7.44	6.15	6.8	6.8	6.8	6.8	6.8	309	9.16	8.42	8.42	546
**5**	1.8	1.8	1.8	1.8	1.8	2.31	1.8	389	4.9	5.92	4.69	490
**2.5**	1.67	1.67	1.67	1.67	1.67	1.67	1.67	418	2.51	2.51	2.51	479
**1**	0.54	0	0.54	0.54	0.54	0.54	0.54	370	0.59	0.59	0.59	505

### Complex mixtures and jumping PCR

**Figure 3:**
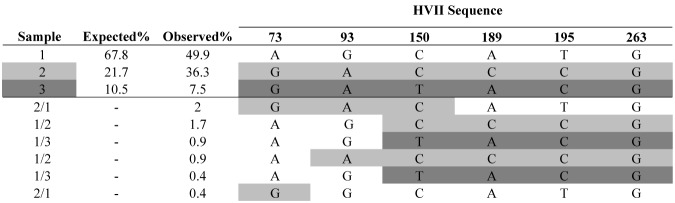
Hypervariable region II (HVII) region sequencing results obtained from a mixture sample composed of three contributors. Three samples differing at multiple base positions were combined (67.8%, 21.7%, and 10.5%) based on mtDNA copy number prior to HVI/HVII amplification. The frequency of the observed sequence reads for the 454 HVII amplicon is reported as determined using the Amplicon Variant Analyzer Software (454 Life Sciences, Bradford, CT, USA). Reads for the HVI and HVII amplicon corresponding to each of the three contributors were observed (results for HVII presented). Further, 6 chimeric sequences were detected, each occurring at low frequency (<2%).

A complex mixture study (≥3 contributors) was conducted to test the 454 HVI/HVII assay’s capability to detect and resolve multiple sequences in a mixed sample. 454 HVII NGS results for a three person mixture are presented in Figure 3. Using the 454 HVI/HVII NGS assay, we were able to detect and resolve each of the three contributor sequences in a complex mixture. The three contributors’ distinct sequences were resolved from a mixture sample, and showed concordance with the Sanger sequencing results at all base substitution positions excluding the homopolymer C-stretch region in HVII (Figure 3). Further, evidence of jumping PCR (*in vitro* crossover artifact) was observed in each of the complex mixtures as chimeric or hybrid sequences were present at low levels (Figure 3). A total of six hybrid HVII sequences were observed ranging from 0.4 to 2% for the three person complex mixture sample.

**Figure 4:**
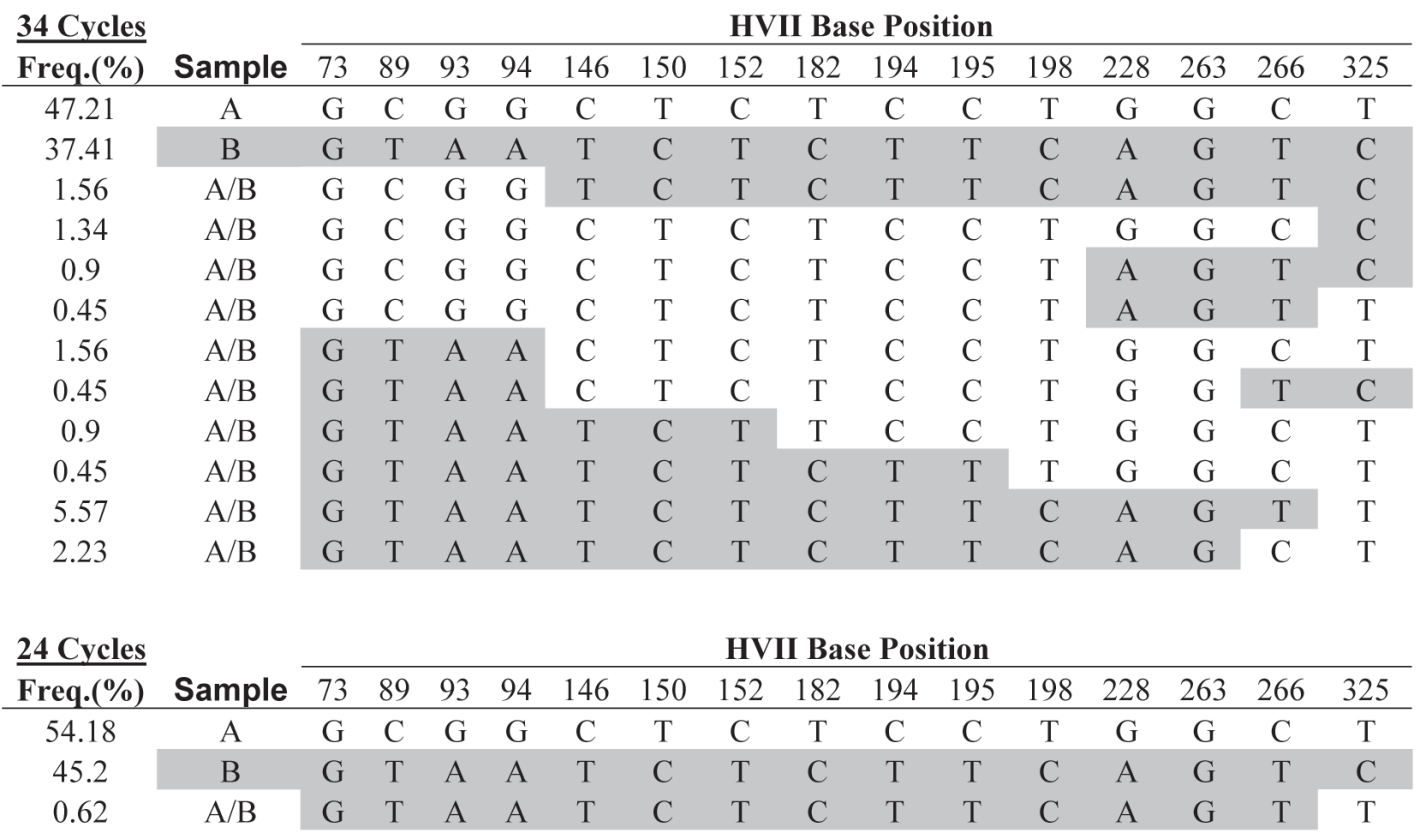
Characterization and reduction of jumping polymerase chain reaction (PCR) artifacts observed in 454 sequencing data. Two different samples (A and B) differing at 15 positions in the hypervariable region II (HVII) were mixed at equimolar mtDNA copies prior to HVI/HVII PCR and amplified at varying cycle numbers – 24,26,28,30,32,34. HVII sequence results obtained using the 454 GS Jr from a sample amplified with 34 cycles and 24 cycles are presented. The number of chimeric sequences (10 sequences) with 34 cycles of amplification decreased to one chimeric sequence when the amplification cycle number was reduced to 24 cycles. Additionally, the observed frequency of chimeric sequences decreased dramatically from 13.18% to 0.62% when the cycle number was reduced.

Jumping PCR, a phenomenon where a single strand of DNA partially extended on one template can act as a primer on the other template to generate an amplicon with chimeric sequences, can occur during late PCR cycles when the number of amplicon copies is very high and the concentration of primers and enzyme is low (27). As expected, an increase in the number and the frequency of chimeric sequences resulting from jumping PCR events was observed with each increase in cycle number from 24 to 34 cycles (Figure 4). The 50:50 mixed sample amplified with 34 cycles resulted in 10 different chimeric sequences with a total frequency of 15.41%. At 24 cycles, one chimeric sequence was detected with a frequency of 0.62% (Figure 4). Chimeric sequences generated from jumping PCR events in mixed samples could be problematic for the forensic application of NGS if they are observed at a high frequency and are not properly recognized. Further improvements to NGS software to include filters to flag or remove jumping PCR chimeras should be considered to aid with mixture analysis.

### Biological mixture sequence application: heteroplasmy

**Figure 5:**
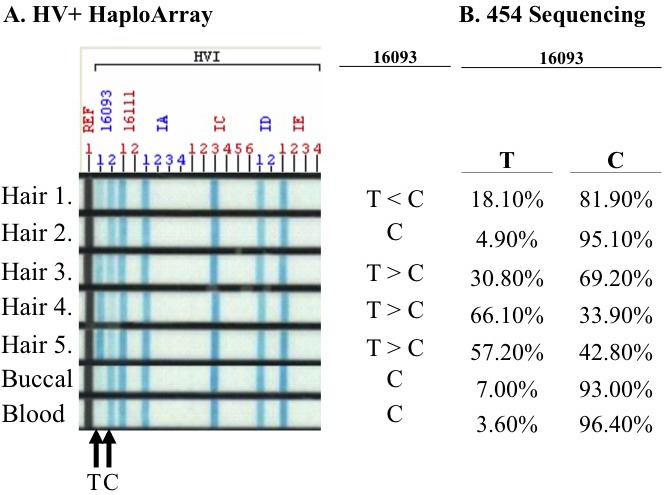
Comparison of methods for detection and quantification of heteroplasmy. Panel A shows the HV+ 5 plex immobilized sequence-specific oligonucleotide probe typing data and Panel B shows the percent reads observed by 454 NGS for each base (T or C) at the heteroplasmic position 16093 for five hair samples, a buccal, and a blood sample from one individual heteroplasmic at a hot spot position 16093 in the hypervariable region I (HVI) region. A single signal corresponding to a C at position 16093 was observed for the blood, buccal, and one of the five hair samples (Hair 2) using the HV+ 5 plex probe panel; heteroplasmy was detected at position 16093 in all samples using the more sensitive 454 NGS technology (average of 750 read depth per sample).

To further determine the detection limits of the 454 HVI/HVII NGS assay, a heteroplasmy study was conducted. HV+ HaploArray (HaploID Genetics, Alameda, CA, USA) and 454 HVI/HVII NGS results from five hairs, blood, and buccal samples from one twin pair with heteroplasmy at position 16093 are presented in Figure 5. HV+ HaploArray analysis detected a mixture of bases (T/C) at position 16093 for hair samples 1,3,4, and 5, observed as two positive probe signals for 16093 (1/2). While relative proportions of the T/C bases can be estimated by the intensity of the probe signal, an observed frequency for each base can be determined for the NGS analysis (Figure 5). The 454 NGS analysis was also more sensitive for the detection of heteroplasmy. NGS detected a mixture of T/C at position 16093 for hair sample 2, blood, and buccal DNA, whereas HV+ HaploArray analysis detected only a C at 16093 (observed as a positive 16093 2 probe signal). The observed frequency of the T base at position 16093 in hair sample 2, buccal, and blood (4.9%, 7.0%, and 3.6%, respectively) was also below the detection limits of Sanger sequencing (typically 10%-15%).

## Discussion

We developed a duplex PCR assay targeting the mtDNA (HVI/HVII) using eight sets of 454 MID-tagged fusion primers in a combinatorial approach for deep sequencing 64 samples in parallel on a 454 GS Jr. Compared with Sanger sequencing of the same samples, the sequence concordance was 100% outside of the homopolymeric regions in HVI and HVII using a 10% cut-off for the 454 sequencing data. Sequencing errors appearing as IN/DEL mutations in homopolymer regions were observed. This issue is a well known feature of NGS based on pyrosequencing (28). Recent publications using the 454 GS Junior and the Ion Torrent PGM instrument, which detects the release of protons rather than pyrophosphate, as in the 454 system, also reported difficulties in sequencing the homopolymeric regions of mtDNA HVI/II (29,30). Since an imbalance in the frequency of the IN/DEL errors was observed between the forward and reverse reads, filters can be applied using software tools, such as NextGene software (SoftGenetics, State College, PA, USA), to identify these sequencing errors in the bioinformatic analysis.

Our assay was shown to be highly sensitive for sequencing limited DNA amounts ( ~ 100 mtDNA copies) and detecting mixtures with low level variants ( ~ 1%) as well as “complex” mixtures (≥3 contributors) not possible to analyze by Sanger sequencing. The minor base was reliably detected at each of the mixed base positions and the observed frequencies were similar to the expected frequencies using 454 sequencing with ~ 600-1000 reads per amplicon. Further, we characterized jumping PCR and resulting hybrid artifact sequences that can arise in amplification of mixed samples and noted that the frequency of the phenomenon was template amount- and cycle-dependent. This effect, which occurs at late cycles of PCR, can be decreased by simply lowering the amplification cycle number. In addition, such chimeric PCR artifacts can be potentially filtered through bioinformatics tools.

The clonal nature of NGS provides a powerful and quantitative means of deconvoluting mixtures, a particularly challenging category of forensics specimens. Multiple contributors to a mixture can be detected by analyzing the different clonal sequence reads identified in the sequence analysis and their contribution quantified by simply counting the number of sequence reads. This digital analysis is much more precise than estimating peak height or area for STR markers or analyzing Sanger electropherograms for mtDNA sequences. For chromosomal markers, one must make assumptions about which alleles in a mixture “go together” as a genotype. Many statistical approaches to mixture analysis consider all possible genotype combinations and, in some cases, different numbers of contributors. Mitochondrial DNA, on the other hand, is a particularly useful marker for deconvoluting mixtures because, potential heteroplasmy aside, each distinct mtDNA sequence corresponds to (at least) one contributor. Statistical analyses can capture and incorporate the probability that one mtDNA sequence could correspond to more than one contributor. Sequencing the entire mtDNA genome rather than the HVI/II regions reported here will provide additional valuable discrimination power and several groups have recently reported NGS analyses using long range PCR (29-31). Recently, we have developed a hybrid capture method of whole mtDNA genome sequencing (32). However, software analyses of whole mtDNA genome sequences from mixed samples remain a challenge. In general, NGS analysis of mtDNA, whether of whole genome or only HVI/II, represents the most robust approach to estimating the number of contributors to a mixture and resolving the mixed DNA sequences.
