# Supplementary material for: Analysis of mixtures using next generation sequencing of mitochondrial DNA hypervariable regions
Source: Croat Med J. 2015 Jun;56(3):208–17. doi: 10.3325/cmj.2015.56.208 (PMC4500979; doi:10.3325/cmj.2015.56.208)
Supplement: Supplementary Table 1 [file CroatMedJ_56_s006.pdf]

**Supplementary Figure 1 (S2.)** Combinatorial approach allows 64 different combinations of MID tagging using eight pairs of primers. The column in bold indicates the MID tag in the reverse primer (grey) while the row in bold indicates the MID tag in the primer forward direction (white).

| **MIDs For/Rev** | **1** | **2** | **3** | **4** | **5** | **6** | **7** | **8** |
|---|---|---|---|---|---|---|---|---|
| **1** | 1 **1** | 1 2 | 1 3 | 1 4 | 1 5 | 1 6 | 1 7 | 1 8 |
| **2** | 2 1 | 2 2 | 2 3 | 2 4 | 2 5 | 2 6 | 2 7 | 2 8 |
| **3** | 3 1 | 3 2 | 3 3 | 3 4 | 3 5 | 3 6 | 3 7 | 3 8 |
| **4** | 4 1 | 4 2 | 4 3 | 4 4 | 4 5 | 4 6 | 4 7 | 4 8 |
| **5** | 5 1 | 5 2 | 5 3 | 5 4 | 5 5 | 5 6 | 5 7 | 5 8 |
| **6** | 6 1 | 6 2 | 6 3 | 6 4 | 6 5 | 6 6 | 6 7 | 6 8 |
| **7** | 7 1 | 7 2 | 7 3 | 7 4 | 7 5 | 7 6 | 7 7 | 7 8 |
| **8** | 8 1 | 8 2 | 8 3 | 8 4 | 8 5 | 8 6 | 8 7 | 8 8 |
