# Supplementary material for: Analysis of mixtures using next generation sequencing of mitochondrial DNA hypervariable regions
Source: Croat Med J. 2015 Jun;56(3):208–17. doi: 10.3325/cmj.2015.56.208 (PMC4500979; doi:10.3325/cmj.2015.56.208)
Supplement: Supplementary Figure 1 [file CroatMedJ_56_s007.pdf]

Table S1. Base Extended Multiplex Identifier (MID) Set Sequences (MID Tags 1-8)

	Sequence
**MID 1**	ACGAGTGCGT
**MID 2**	ACGCTCGACA
**MID 3**	AGACGCACTC
**MID 4**	AGCACTGTAG
**MID 5**	ATCAGACACG
**MID 6**	ATATCGCGAG
**MID 7**	CGTGTCTCTA
**MID 8**	CTCGCGTGTC
